# The Non-Legume *Parasponia andersonii* Mediates the Fitness of Nitrogen-Fixing Rhizobial Symbionts Under High Nitrogen Conditions

**DOI:** 10.3389/fpls.2019.01779

**Published:** 2020-02-07

**Authors:** Simon E. Dupin, René Geurts, E. Toby Kiers

**Affiliations:** ^1^ Department of Ecological Science, Vrije Universiteit Amsterdam, Amsterdam, Netherlands; ^2^ Department of Plant Sciences, Wageningen University, Wageningen, Netherlands

**Keywords:** *Parasponia*, nitrogen fixing bacteria, plant nutrition, host control, rhizobium fitness, nitrogen fertilizer, non-legume, nodulation

## Abstract

Organisms rely on symbiotic associations for metabolism, protection, and energy. However, these intimate partnerships can be vulnerable to exploitation. What prevents microbial mutualists from parasitizing their hosts? In legumes, there is evidence that hosts have evolved sophisticated mechanisms to manage their symbiotic rhizobia, but the generality and evolutionary origins of these control mechanisms are under debate. Here, we focused on the symbiosis between *Parasponia* hosts and N_2_-fixing rhizobium bacteria. *Parasponia* is the only non-legume lineage to have evolved a rhizobial symbiosis and thus provides an evolutionary replicate to test how rhizobial exploitation is controlled. A key question is whether *Parasponia* hosts can prevent colonization of rhizobia under high nitrogen conditions, when the contribution of the symbiont becomes nonessential. We grew *Parasponia andersonii* inoculated with *Bradyrhizobium elkanii* under four ammonium nitrate concentrations in a controlled growth chamber. We measured shoot and root dry weight, nodule number, nodule fresh weight, nodule volume. To quantify viable rhizobial populations *in planta*, we crushed nodules and determined colony forming units (CFU), as a rhizobia fitness proxy. We show that, like legumes and actinorhizal plants, *P. andersonii* is able to control nodule symbiosis in response to exogenous nitrogen. While the relative host growth benefits of inoculation decreased with nitrogen fertilization, our highest ammonium nitrate concentration (3.75 mM) was sufficient to prevent nodule formation on inoculated roots. Rhizobial populations were highest in nitrogen free medium. While we do not yet know the mechanism, our results suggest that control mechanisms over rhizobia are not exclusive to the legume clade.

## Introduction

Symbiotic partnerships have transformed the Earth’s nutrient cycles and facilitated rapid adaptation of species to divergent new niches (Joy, 2013; McFall-Ngai et al., 2013; Oldroyd, 2013). Because almost all organisms rely on symbiotic associations for some form of metabolism, protection, or energy (Bronstein, 2015), this immense symbiotic microbial world has been called “the biggest frontier that biology has presented us in a long time” (Carey, 2015).

Despite their importance, understanding the origins and evolutionary trajectories of symbiotic partnerships remains a major challenge. The problem is that mutual benefit does not guarantee evolutionary stability, and partnerships can be vulnerable to exploitation (Sachs and Simms, 2006; Kiers and Denison, 2008; Sachs et al., 2011; Sachs and Hollowell, 2012). What prevents microbial mutualists from defecting from symbiotic cooperation and parasitizing their hosts? This is a question being asked across a diversity of host organisms, from plants and insects to humans (Werner et al., 2014; Hallam and McCutcheon, 2015; Keeling et al., 2015; Werner et al., 2015). While it is appreciated that symbiosis is a key underlying mechanism behind the complexity of life, we do not have a general understanding of how symbiotic associations are controlled and harnessed (Keeling et al., 2015).

The legume-rhizobia N_2_ fixing symbiosis has become an emerging model system in evolutionary biology to study host control (Kiers et al., 2006; Kiers et al., 2007; Oono et al., 2009; Porter and Simms, 2014; Regus et al., 2015; Westhoek et al., 2017; Porter et al., 2019). Host plants employ a range of (non-exclusive) strategies to maximize rhizobial benefits, including: 1) pre-nodule control in which the plant evolved high levels of specificity to achieve species, or even strain-specific selection on rhizobial partners, and 2) control based on rhizobial performance, such that higher performing nodules receive proportionally more resources than poor performing nodules (Kiers and Denison, 2008; Westhoek et al., 2017). Because signaling (i.e., pre-nodule control) can be vulnerable to partners that cheat by evolving the correct signal while providing few resources (Edwards and Yu, 2007), it is thought that some form of basal nodule-level control is necessary to prevent rhizobial exploitation (Denison and Kiers, 2011; Regus et al., 2017a; Kiers et al., 2003; Kiers et al., 2006; Oono et al., 2011).

Control of exploitation is particularly important when plant hosts have direct access to high levels of nitrogen in the soil. Under these conditions, the nitrogen benefits provided by the rhizobial symbiont become redundant with nitrogen provided by the environment. Hosts are therefore expected to evolve mechanisms which prevent nodule formation when grown in high nitrogen soils (Regus et al., 2017b). While such regulatory pathways have been documented in legume species (Streeter and Wong, 1988; Cabeza et al., 2014; Soyano et al., 2014; Nishida et al., 2016; Nishida and Suzaki, 2018), the generality of these control mechanisms are unknown (Heath et al., 2010; Regus et al., 2014; Regus et al., 2015).

Our aim was to study host control mechanisms outside the legumes. We focused on the non-legume *Parasponia andersonii* (Cannabaceae). The genus *Parasponia* is composed of pioneer plant species typically found on nitrogen poor slopes of volcanic hills in the Malay Archipelago. *Parasponia* is the only lineage outside the legume family to be able to form a nodule symbiosis with rhizobium (Trinick, 1973; Trinick and Galbraith, 1980; Trinick and Hadobas, 1988). Recent phylogenomic studies suggest that nodule symbioses with diazotrophic bacteria share a single evolutionary origin (Griesmann et al., 2018; van Velzen et al., 2019). As the *Parasponia* and legumes diverged >100 million years ago, microbial partner selection strategies were shaped independently in both lineages (van Velzen et al., 2019). Therefore, *Parasponia* is a unique evolutionary replicate of rhizobium symbiosis that allows us to better understand the evolutionary origins of control mechanisms.

In legumes, such *Glycine max*, past work has shown that the severity of symbiont control is mediated by the addition of external nitrate—as nitrogen availability increases, the host reduces resources allocated to the symbiont. Because soil nitrate is generally less costly for legumes compared to biologically fixed nitrogen, this leads to an inhibition or severe reduction of legume nodule formation (Streeter and Wong, 1988; Voisin et al., 2002; Wendlandt et al., 2018; Regus et al., 2017b). This process, however, is not well understood in non-legumes. In *Parasponia*, early work has shown nodules can continue to form in high nitrogen environments (Becking, 1983b), but whether rhizobia remain viable in nodules under high nitrogen conditions is unknown. This is important because in *Parasponia* nodules, rhizobium are not terminally differentiated (Alunni and Gourion, 2016). This means that the endosymbiont population within nodules can replicate, and will be added to the soil population upon nodule senescence. Can non-legumes control symbionts under conditions when the symbionts become a cost rather than a benefit? Studying the existence of control patterns has been particularly challenging in *Parasponia* nodules due to the difficulty of growing these tropical trees in greenhouses, and the small size of their nodules compared to most model legumes. Typical metrics, such as growth parameters of individual nodules, poly-3-hydroxybutyrate (PHB) content, and rhizobial fitness measures, have been historically difficult to obtain.

Here we study the effects of increasing nitrogen fertilization on the symbiosis between *P. andersonii* and the rhizobial symbiont *Bradyrhizobium elkanii*. Our aim was to ask if fertilization: 1) reduced or eliminate the growth benefits of rhizobial nodulation for *P. andersonii*, and 2) reduced the fitness benefits for the rhizobial symbiont. If *Parasponia* has evolved effective mechanisms to control nodulation under high nitrogen, then we expect increasing fertilization to be correlated with negative fitness consequences for rhizobia. If these mechanisms are costly for the host to enact, then we expect to see a host growth depression in the presence—but not absence—of a rhizobial symbiont under the high nitrogen treatments.

We grew plants under 0, 0.0375, 0.375, 3.75 mM ammonium nitrate concentrations, either with or without rhizobial inoculation. Our four levels were chosen to represent specific ecological challenges for the host-symbiont, namely: i) when *Parasponia* depends entirely on its symbiont for nitrogen input (0 and 0.0375 mM), ii) when benefits from inoculation are minimal (0.375 mM), iii) when benefits from inoculation are absent/negative (3.75 mM). After 4 weeks, we quantified shoot and root dry weight, nodule number, and nodule fresh weight. We developed an imaging protocol to measure the projected surface of individual nodule areas and then converted it to volume as a second metric for rhizobia benefit. We crushed nodules for measures of colony forming units (CFU) to determine rhizobial populations per nodule. Together, these metrics (nodule number, biomass, volume, and CFUs) provided us with a proxy for *in planta* rhizobial fitness that allowed us to better understand host control in non-legumes.

## Materials and Methods

### Seed Germination

We harvested fresh berries from *in-vitro* propagated *Parasponia andersonii* trees genotype W1-14 (Van Velzen et al., 2018) maintained in a tropical greenhouse. We extracted the seeds from the berries by soaking them in water and gentle rubbing against a fine sieve. We surface sterilized all seeds in 4% sodium hypochlorite for 15 min and washed seven times with sterile MQ water. We induced germination by temperature cycle (4 h 28°C, 4 h 7°C) for 12 days. We incubated seed on Schenk and Hildebrandt medium agar plates for 10 days until cotyledons were fully emerged.

### Experimental Design and Plant Growth

We prepared the growing medium of sterile perlite and sterile river sand. We added 210 g of each perlite mixture to 10 sterile polypropylene containers (OS140box, Duchefa Biochemie) allowing for better gas exchange. Per pot, we placed 4 cm^3^ of the river sand mixture to transfer the seedling and avoid root desiccation. We used a factorial design experiment consisting of two rhizobia conditions, four nitrogen levels, and 10 replicates per treatment (2 x 4 x 10 = 80 pots).

### Inoculation and Nitrogen Treatments

To inoculate *P. andersonii*, we chose the highly efficient nodulating strain *B. elkanii* WUR3 (Op den Camp et al., 2012). To prepare the inoculum, we grew a WUR3 pre-culture from a single colony in liquid peptone-salts-yeast (PSY) medium at 28°C, 60 rpm (Regensburger and Hennecke, 1983). One milliliter of the pre-culture OD_600_ = 0.8 was used to inoculate a 200 ml Erlenmeyer culture. We harvested the culture by centrifugation [10 min at 3,500 x relative centrifugal force (rcf)] at OD_600_ = 0.8. We suspended the cells in the different EKM (Becking, 1983a) solutions to an OD_600_ = 0.05. The nitrogen treatments were based on an EKM-medium with four levels (0, 0.0375, 0.375, and 3.75 mM) ammonium nitrate. Nitrogen and rhizobia inoculum were added by saturating the perlite and river sand with the four EKM-medium prior to transferring the seedlings to pots. Non-inoculated controls received EKM solutions (see below) but without the rhizobia culture. We then randomly placed the pots on a growth chamber table under a 16/8 h light cycle, temperature 26/24°C, light intensity 185 µmol/m2/s, and a relative humidity of 90%.

### Harvest

We harvested plants after 30 days. We carefully washed off the perlite and sand from the root systems. We counted nodules and harvested each one individually. We then used binoculars equipped with a Nikon camera (DS–Fi2) to image each nodule. To obtain nodule volume, we extracted and measured the area and perimeter of the nodules photographed using FIJI (Schindelin et al., 2012). We calculated the corresponding prolate spheroid volume using the best fitted ellipse of each nodule based on a previously developed formula (Nedomová et al., 2014). We weighed nodules and kept them at 4°C in 0.9% NaCl solution until they could be crushed for fitness assays. We separated shoots and roots, dried them 72 h at 60°C, and weighed them.

### Metrics for Rhizobial Fitness Proxies

To determine a fitness proxy for rhizobia per plant, we surface sterilized all nodules with 96% ethanol for 20 s, 4% sodium hypochlorite for 1 min, and washed seven times with sterile water. We crushed nodules in 150 µl 0.9% sterile saline solution. Fifty microliters of the crushed nodules was diluted in series and both 10,000 and 100,000 dilutions were streaked on PSY plates with sterile glass beads and incubated at 28°C for 7 days. We then counted colonies to determine total rhizobia per plant.

### Statistical Analysis

We used R version 3.6.0 (2019-04-26) to conduct all statistical tests. In case of heteroscedasticity or non-normality, a decimal logarithm transformation of the data was performed to meet ANOVA assumptions. If ANOVA assumption could not be met with a transformation of the response variables, a non-parametric Kruskal test was conducted. To compare plant biomass among treatments, we tested the decimal logarithm mean plant dry biomass for significant differences with two-way ANOVA and a *post-hoc* Tukey tests for pairwise comparison with a 95% confidence interval. To test for differences in allocation to above and belowground parts, we compared mean root to shoot ratio with a pair-wise Wilcoxon test. To compare plant biomass as a function of rhizobial inoculation, we compared decimal logarithm relative plant biomass among nitrogen treatments with a one way ANOVA and a Tukey test for multiple group comparison with a 95% confidence interval. To test for differences in nodule formation, we compared mean nodule number and nodule fresh per plant with one-way ANOVA and a Tukey test for multiple group comparison with a 95% confidence interval. For nodule volume we used the Kruskal test and Dunn’s *post-hoc*. Rhizobial fitness components, defined as viable rhizobia per milligram of plant, nodule mass, or volume, were compared among the four nitrogen treatments of inoculated plants with Kruskal test and Dunn’s *post-hoc*. Dunn’s test is appropriate for groups with unequal numbers of observations (Zar, 2010), and was corrected for multiple comparisons following Benjamini and Hochberg method (1995) with a 95% confidence interval.

## Results

### Plant Biomass

**Figure 1:**
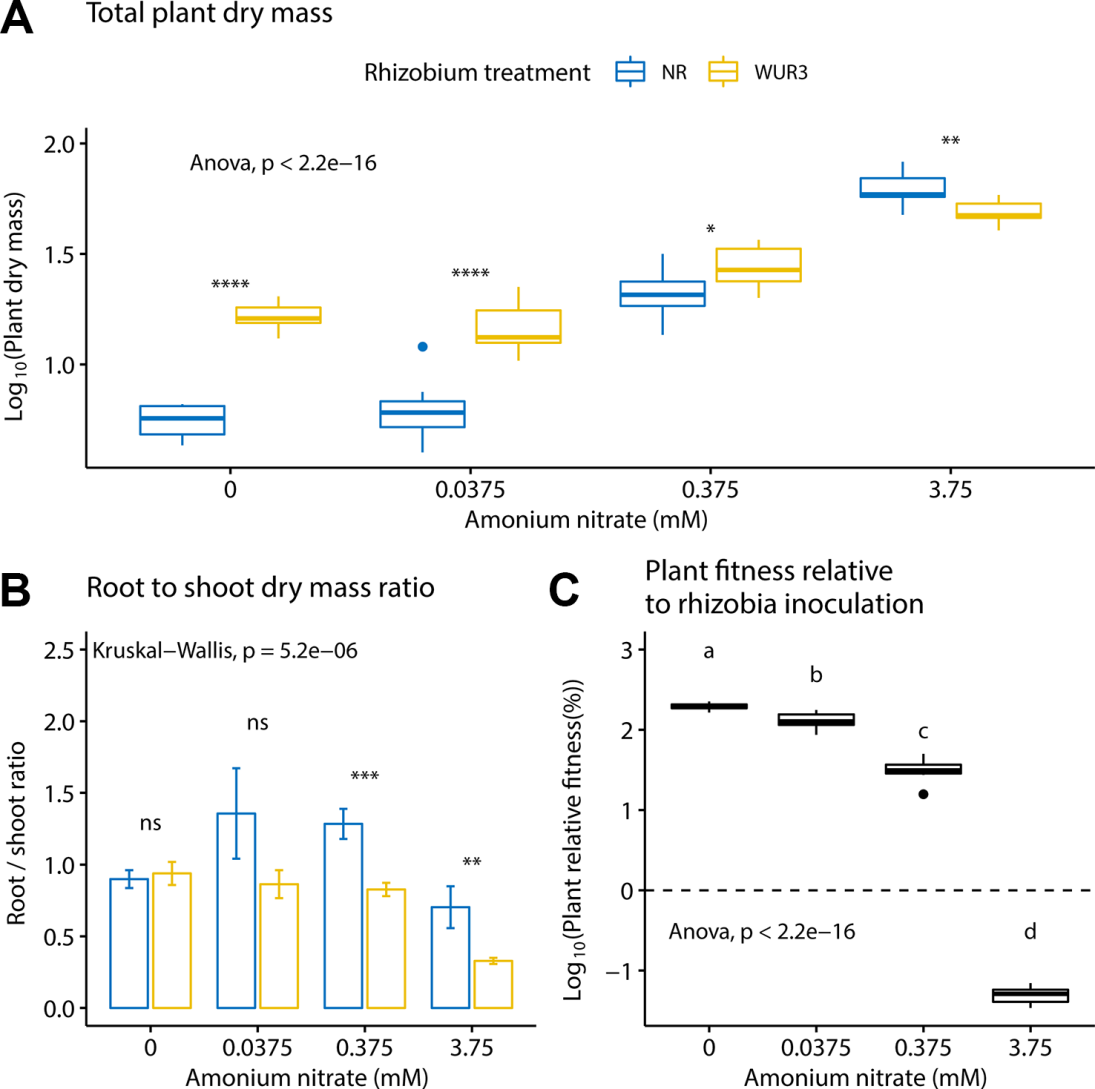
Inoculated and non-inoculated *Parasponia andersonii* plant mass under four nitrogen levels. **(A)** Mean total (shoot + root) plant dry weight. Asterisks show pair-wise comparison significance for each nitrogen level [ANOVA, Tukey honest significant difference (HSD) test]. **(B)** Mean root to shoot dry weight ratio. Asterisks show pair-wise comparison significance for each nitrogen level (Wilcoxon rank sum test) NR = non-rhizobial (blue), WUR3 = Rhizobial strain B. elkanii WUR3 (yellow). **(C)** Mean relative host growth response to rhizobia inoculation as the difference of the total dry weight of inoculated and non-inoculated control, divided by the total dry weight of non-inoculated control. Letters represent groups significantly different from each other (ANOVA, Tukey HSD test). Error bars show standard error.

We first asked how increasing soil nitrogen affected plant growth patterns in the presence and absence of rhizobial symbionts. We found that nitrogen treatment and rhizobial treatment both had a significant effect on total plant biomass (ANOVA; F_3,72_ = 330.6, p < 0.001 and F_1,72_ = 112.5, p < 0.001 respectively), with a significant interaction term between the two variables (ANOVA; F_3,72_ = 40.2, p < 0.001, **Figure 1A**). Specifically, we found the presence of rhizobia increased total plant biomass at 0, 0.0375, and 0.375 mM ammonium nitrate levels. At the highest nitrate level (3.75 mM), we found that the presence of rhizobia became a cost (**Figure 1A**). By quantifying the root to shoot ratio across these nitrogen levels, we found that at the highest nitrate level, the rhizobial cost was related to a decrease in root biomass (**Figure 1B**).

To assess plant biomass relative to rhizobia inoculation under different nitrogen regime, we calculated the relative growth benefit of inoculation for the plant (**Figure 1C**). Here, we took the difference of the total dry weight of the size-matched inoculated and non-inoculated control, divided by the total dry weight of non-inoculated control. We found that the relative growth benefits of rhizobial inoculation decreased with nitrogen fertilization (ANOVA; F_3,36_ = 2,685, p < 0.001).

### Formation of Symbiotic Organs

**Figure 2:**
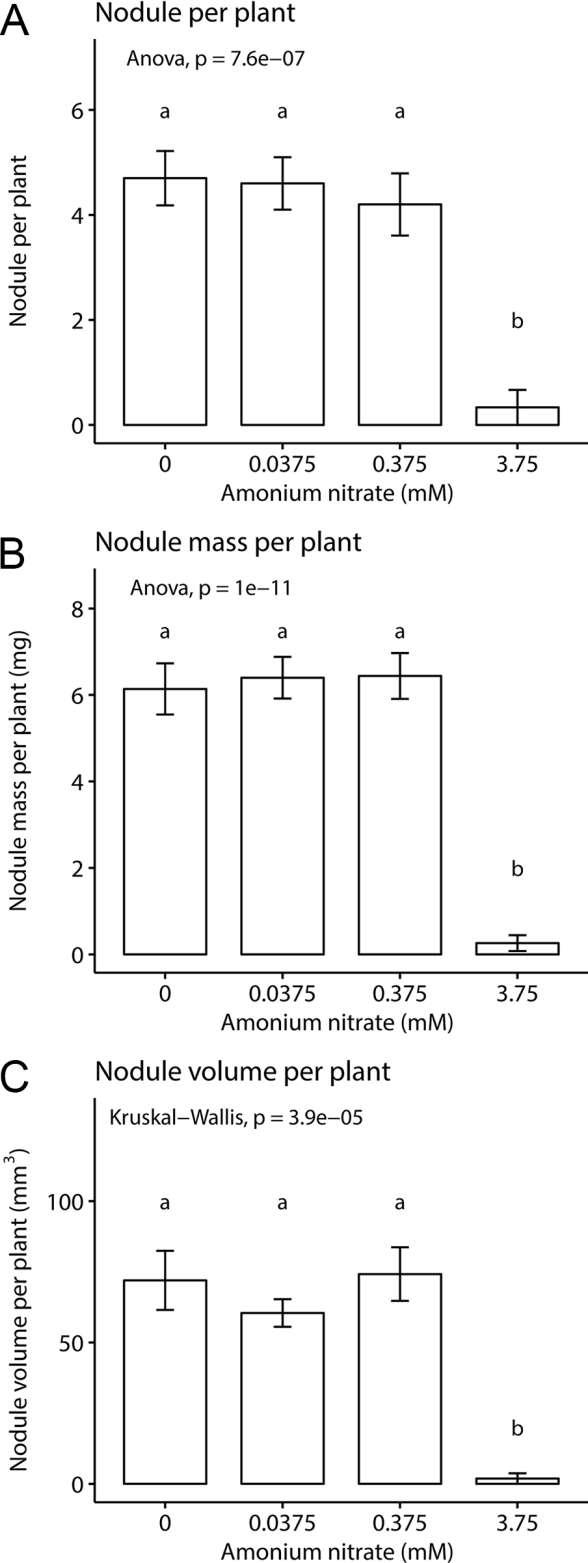
Inoculated *Parasponia andersonii* nodulation under four nitrogen levels. **(A)** Mean nodule number per plant. **(B)** Mean nodule fresh weight per plant. **(C)** Mean nodule volume per plant. Letters represent groups significantly different from each other (ANOVA, Tukey HSD test and Kruskal test, Dunn’s test). Error bars show standard error.

We then asked how nitrogen fertilization affected the formation of symbiotic organs, namely nodule number, total nodule fresh weight, and nodule volume. We found a significant effect of fertilization on all three parameters: nodule number (one-way ANOVA; F_3,35_ = 16.5, p < 0.001) nodule weight (one-way ANOVA; F_3,36_ = 41.08, p < 0.001), and nodule volume (Kruskal-Wallis test; chi-squared = 23.07, df = 3, p-value < 0.001) (**Figure 2**). This effect was driven largely by the highest nitrogen concentration. In the three lowest nitrogen concentrations, we found no difference in nodule number (~4–5 per plant, **Figure 2A**), fresh weight (~6 mg, **Figure 2B**), or volume (~70 mm^3^, **Figure 2C**). However, when fertilization was increased to 3.75 mM NH_4_NO_3_, all nodule parameters were reduced to nearly zero, across all replicates. This demonstrates that with enough exogenous nitrogen available, *P. andersonii* is able to prevent nodule organogenesis, similar as reported for legumes and actinorhizal plants.

In legumes, it has been demonstrated that the rhizobia—as well as exogenous fixed-nitrogen—can trigger expression of CLE peptide encoding genes, which can trigger systemic signalling and inhibit nodulation (Okamoto et al., 2009; Reid et al., 2011a). Studies in *Lotus japonicus* revealed that whereas *LjCLE-RS1* and *LjCLE-RS2* are induced upon rhizobium inoculation, only *LjCLE-RS2* is induced by application of exogenous nitrogen (Okamoto et al., 2009). The latter gene is a close homolog of the soybean (*G. max*) CLE peptide encoding gene *GmNIC1a* that is induced by exogenous nitrate (Reid et al., 2011b; Hastwell et al., 2017). For *P. andersonii*, *CLE* genes have been annotated (**Table S1**). Of these, *PanCLE5* and *PanCLE9* showed to be close homologs of *LjCLE-RS1* and *LjCLE-RS2*, and their counterparts *MtCLE12* and *MtCLE13* in *Medicago truncatula* (Van Velzen et al., 2018). To obtain insight whether any of the *P. andersonii CLE* genes is induced by exogenous nitrate, we exploited available RNA sequencing (RNA-seq) data (Van Velzen et al., 2018). This revealed that three *CLE* genes, namely *PanCLE2*, *PanCLE8*, and *PanCLE9,* have an increased expression in inoculated roots grown at relatively high exogenous nitrate levels (5 mM KNO_3_) (**Figure S1**). These three genes showed a similar induction in young, non-infected, nodule primordia (**Figure S1**). This suggests that in *P. andersonii* rhizobium and exogenous nitrate trigger an overlapping *CLE* gene repertoire to regulate nodulation.

### Rhizobial Fitness Proxies

**Figure 3:**
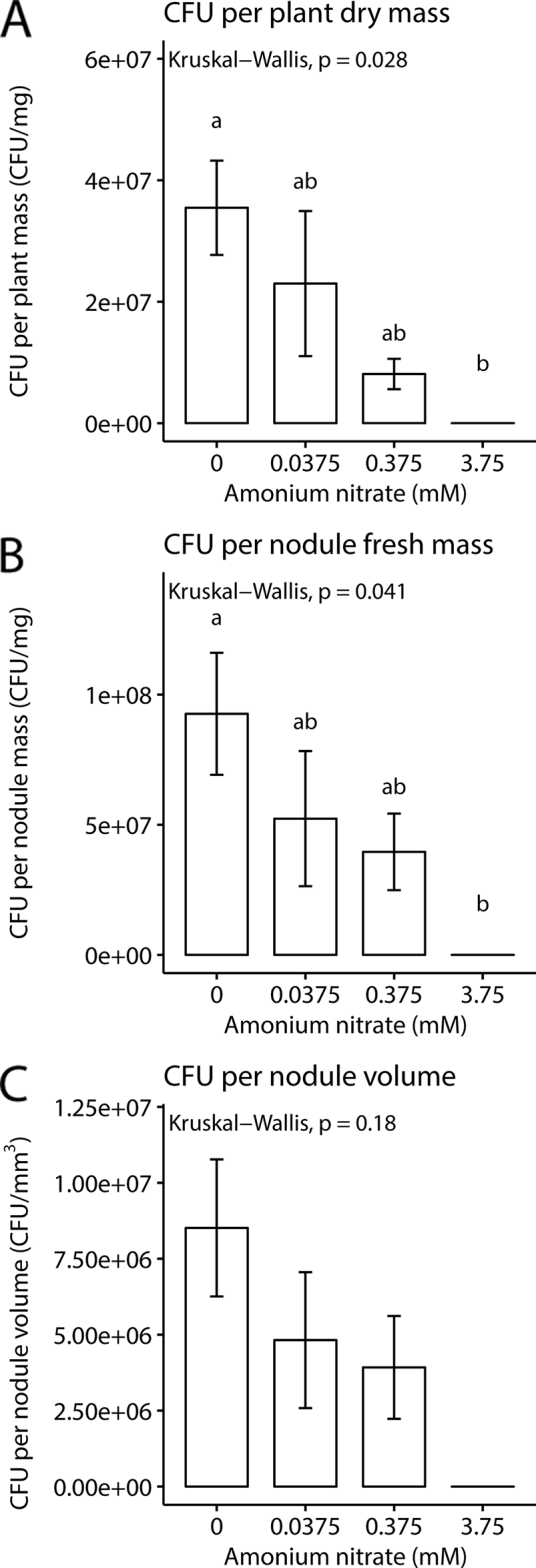
Rhizobia fitness under four nitrogen levels. **(A)** Mean colony forming units per mg plant dry weight. **(B)** Mean colony forming units per milligram nodule fresh weight. **(C)** Mean colony forming units per volume of nodule. Letters represent groups significantly different from each other (Kruskal test, Dunn’s test). Error bars show standard error.

While nodule number and weight can give some rough estimates of rhizobial benefit, a key parameter is to directly quantify rhizobial densities in nodules. We therefore next measured CFUs per mg of plant and nodule biomass or volume (n = 2 for 3.75 mM as only two plants formed nodules). We found that as exogenous nitrogen levels increased, there was a concurrent decrease in CFUs per plant biomass (Kruskal-Wallis test; chi-squared = 9.1, df = 3, p-value = 0.028), and CFUs per mg of nodule (Kruskal-Wallis test; chi-squared = 8.2, df = 3, p-value = 0.041). But surprisingly, no significance difference was found for CFUs per nodule volume (Kruskal-Wallis test; chi-squared = 4.9, df = 3, p-value = 0.18). Despite the fact that nodule number and fresh weight remained the same across the lowest three nitrogen levels, we documented a decrease in viable rhizobial density in nodules as nitrogen levels increased (**Figure 3**).

## Discussion

Plant species found in the nitrogen fixing clade rely on diazotrophic symbionts to acquire nitrogen in poor soils (McKey, 1994; Crews, 1999; Vitousek et al., 2002). The ability to control resource allocation to these symbionts is likely a key requirement for evolutionary maintenance of the symbiosis (Werner et al., 2015). We found evidence that the non-legume *P. andersonii* has likewise evolved mechanisms to control rhizobial fitness, despite an independent evolutionary trajectory from the legume lineage of >100 million years ago. Specifically, we found that increasing nitrogen levels lead to a decrease in rhizobial populations within nodules. In contrast, evidence for control of nodule formation (i.e., organogenesis) was only evident from the highest nitrogen level, in which nodule formation was almost completely suppressed. In the lower nitrogen treatments, we found no evidence for differences in nodule size, number, or volume. Together, these data suggest that despite the continued growth and formation of nodules at lower nitrogen levels, the host still can control the success of the rhizobial populations within those nodules.

An open question is whether *Parasponia* nodulation is somehow less advanced than in legumes (Behm et al., 2014): our results point to similar levels of nodulation control as found in some legume species. Specifically, it has been shown that at high nitrogen levels, legumes can control nodule formation and mass—a process known as autoregulation of nodulation (Cho and Harper, 1991; Sueyoshi et al., 2003; Jeudy et al., 2010). This has also been demonstrated in nodulated actinorhizal plants, which likewise show a reduced investment in nodulation with application of exogenous nitrogen (Kohls and Baker, 1989; Thomas and Berry, 1989; Arnone et al., 1994; Markham and Zekveld, 2007; Wall and Berry, 2007). Our findings are therefore in line with the idea that a shared mechanism to control nodulation investment across the nitrogen fixing clade was in place before the evolutionary diversion of the nodulating lineages.

How does such control operate? New work has shown that legumes employ a nitrate response to inhibit rhizobial symbiosis by upregulating specific transcription factors (Nishida et al., 2018). Autoregulation of nodulation works as a negative feedback from the key transcription factor NIN targeting CLE peptides, which induces a shoot response, production of cytokinin and inhibition of nodulation (Nishida and Suzaki, 2018). The nitrate-induced inhibition induces the NIN-like transcription factor NRSYM1 targeting the same CLE peptides. Furthermore, in the actinorhizal plant *Casuarina glauca*, RNA interference (RNAi) NIN knockdown also showed the essential role of NIN in controlling nodule formation (Clavijo et al., 2015). While it is unknown if *Parasponia* employs these same mechanisms, CLE peptides are expressed in *P. andersonii* nodules (**Figure S1**), suggesting it could be the case (Van Velzen et al., 2018).

However, the ability to control resource allocation to nodules may be less important to *Parasponia* hosts, given the extremely nutrient poor soils in which they are typically found (Achmad and Hadi, 2016). In this ecological niche, we would not expect hosts to be exposed to high nitrogen conditions, nor the concurrent selection pressures against nodulation. There is also evidence that host responses to nitrate may evolve differently across different plant lineages. Studies of the legume *Acmispon strigosus* found increases in nodule number and size at low nitrate levels, as expected, but also revealed that nodulation suppression was linked with high plant mortality suggesting a high, direct sensitivity to nitrate (Regus et al., 2017b). In *L. japonicus*, fertilization reduced nodule size and nodule number, but with no apparent cost on plant fitness (Nishida et al., 2018). In actinorhizal plant lineages closely related to *P. andersonii*, added nitrate reduces and blocks nodule formation of the *Frankia* symbiosis. In a split root experiment, *Casuarina cunninghamiana* showed localized control depending on exogenous nitrate concentration (Kohls and Baker, 1989; Arnone et al., 1994). In *Parasponia*, nodule size and number did not vary with nitrogen concentration. Instead, nodulation was nearly eliminated for the highest level of fertilization.

Our data show that *P. andersonii* can block nodulation, but that this might entail a cost. We found that at the highest nitrogen level, plant root biomass was reduced in the presence of rhizobia compared to non-inoculated controls. But this growth depression was not observed at lower nitrogen concentrations. Moreover, we observed a linear decrease in the benefit to plant growth conferred by rhizobia as nitrogen increased. Specifically, our data show that resource allocation to root growth was negatively impacted under high N when rhizobia were present (**Figure 1B**). This cost in high nitrogen context was not linked with allocation to resources to nodules organogenesis because nodule formation was suppressed. Instead, this may be linked to the presence of intercellular bacteria, nutrient uptake, or plant defense mechanisms (Bakker et al., 2018). A similar result was also found in *A. strigosus,* whereby the authors noted a cost to the presence of rhizobia under high nitrogen, even though the cost was also not linked to nodule formation (Regus et al., 2017b). They point to past work showing the cost of chemically induced plants defense response to pathogens (Heil et al., 2000). Whether our reduction in root growth (**Figure 1B**) is linked to the costs of upregulating plant defense response is unknown.

Although our data suggest that *Parasponia* employs a mechanism to control nodulation upon presence of exogenous nitrogen—and that this is likely linked to CLE signaling—we also find that rhizobial density may be likewise regulated within nodules. Specifically, we found that *P. andersonii* controlled rhizobial colonization levels at a per nodule milligram and per plant milligram basis. While plants had similar numbers of nodules and equivalent nodule weights across three nitrogen levels, we found that the amount of viable rhizobia hosted in host cells varied. Control of rhizobial fitness within nodules has been shown in legumes, most recently in *L. japonicus,* in which there is evidence that plants can differentially control fitness of effective and ineffective rhizobia within a single nodule (Quides et al., 2017). Similarly, sanction strength—meaning the ability to control rhizobial fitness in individual nodules—was also predicted (West et al., 2002) and shown (Kiers et al., 2006) to increase with addition of external nitrate in soybeans.

While our data suggest that within nodule regulation of rhizobial fitness depends on nitrogen levels, a broader question is whether *Parasponia* has evolved a similar response to ineffective rhizobia that fail to provide nitrogen. Increasingly, work has shown that allocation to nodules will depend on the quality of the rhizobial partners, such that nodules containing low-quality partners will be sanctioned, and experience a reduction in resources (Regus et al., 2017a; Kiers et al., 2003; Oono et al., 2009; Oono et al., 2011). For example work in *Lupinus arboreus* has shown that the size of the nodule is linked with the quality of its occupant (Simms et al., 2006). While we used a well-characterized effective strain, future work should aim to understand how rhizobial partner quality and nitrogen levels interact in the *Parasponia*-rhizobia symbiosis, studied in some legumes (Grillo et al., 2016; Regus et al., 2017a; Regus et al., 2014; Wendlandt et al., 2018).

A second open question is how the physiology of *Parasponia* nodules affects the potential for hosts to control rhizobial fitness. *P. andersonii* has indeterminate nodule with a central vasculature, meaning a meristem sustains a continuous growth of the nodule such that cells become colonized with infection threads containing rhizobia (Behm et al., 2014). When the cells are fully colonized, the rhizobia cells are kept in fixation threads and do not differentiate to bacteroids (i.e., swollen bacteria unable of cell division) (Trinick and Galbraith, 1976). Because of this mode of growth, we had expected *P. andersonii* to reduce nodule growth (meristematic cell division) upon fertilization, yet we observed similar nodule size with an overall lower cell colonization by rhizobia. While the mechanisms is still unknown, this result suggests that *P. andersonii* can directly reduce rhizobia cell division within its nodules or reduce rhizobia nodule occupancy by inducing cell senescence.

More generally, future work is needed to better characterize the costs and benefits of the symbiosis physiologically. For example, from the host side, measurements of %Ndfa (nitrogen derived from the atmosphere) can help us more accurately understand the contribution of nitrogen fixation under different fertilizer regimes. Likewise, detailed microscopy of *P. andersonii* nodules could be conducted. Here, electron microscopy would be useful to study the integrity of the fixation threads and endosymbiotic bacteria, whereas light microscopy on replicate nodules could help develop reliable quantifying techniques based on visual inspection [as in (Regus et al., 2017a)]. From the symbiont side, quantification of metrics such as (PHB), could be helpful in understanding rhizobial fitness, specifically how PHB is linked to reproduction and survival during starvation [e.g., (Ratcliff et al., 2008)].

Overall our work suggests that rhizobial control mechanisms are not exclusive to legumes. While there was evidence that the relative host growth benefits of inoculation decreased with nitrogen fertilization, we found that *Parasponia* controls rhizobial fitness, likely by mediating rhizobial density, depending on ammonium nitrate availability. A key open question is how these processes operate in the field, where *Parasponia* evolved on nutrient poor volcanic soils. Given increasing global nutrient inputs, even to pristine ecosystems, more data are needed to understand fitness alignment in the *Parasponia*—rhizobium symbiosis under changing nutrient conditions.

## Data Availability Statement

The datasets generated for this study are available on request to the corresponding author.

## Author Contributions

All authors contributed to frame the research question and design of the experiments. SD performed the experimental work. All authors contributed to write the manuscript and participated in the discussion.

## Funding

This work was supported by NWO open competition grant to ETK (819.01.007).

## Conflict of Interest

The authors declare that the research was conducted in the absence of any commercial or financial relationships that could be construed as a potential conflict of interest.

## Supplementary Material

Figure S1 Expression profile of *P. andersonii* CLE peptide encoding gene. Expression profile of *P. andersonii* CLE genes in non inoculated roots, nodule promordia and inoculated roots under low (0.5 mM KNO_3_) and high nitrate (5.0 mM KNO_3_) conditions. Expression is given in DESeq2-normalized read counts; error bars represent SE of three biological replicates. Dots represent individual expression levels.
