# Supplementary figures and images for: The Non-Legume Parasponia andersonii Mediates the Fitness of Nitrogen-Fixing Rhizobial Symbionts Under High Nitrogen Conditions
Source: Front Plant Sci. 2020 Feb 7;10:1779. doi: 10.3389/fpls.2019.01779 (PMC7019102; doi:10.3389/fpls.2019.01779)

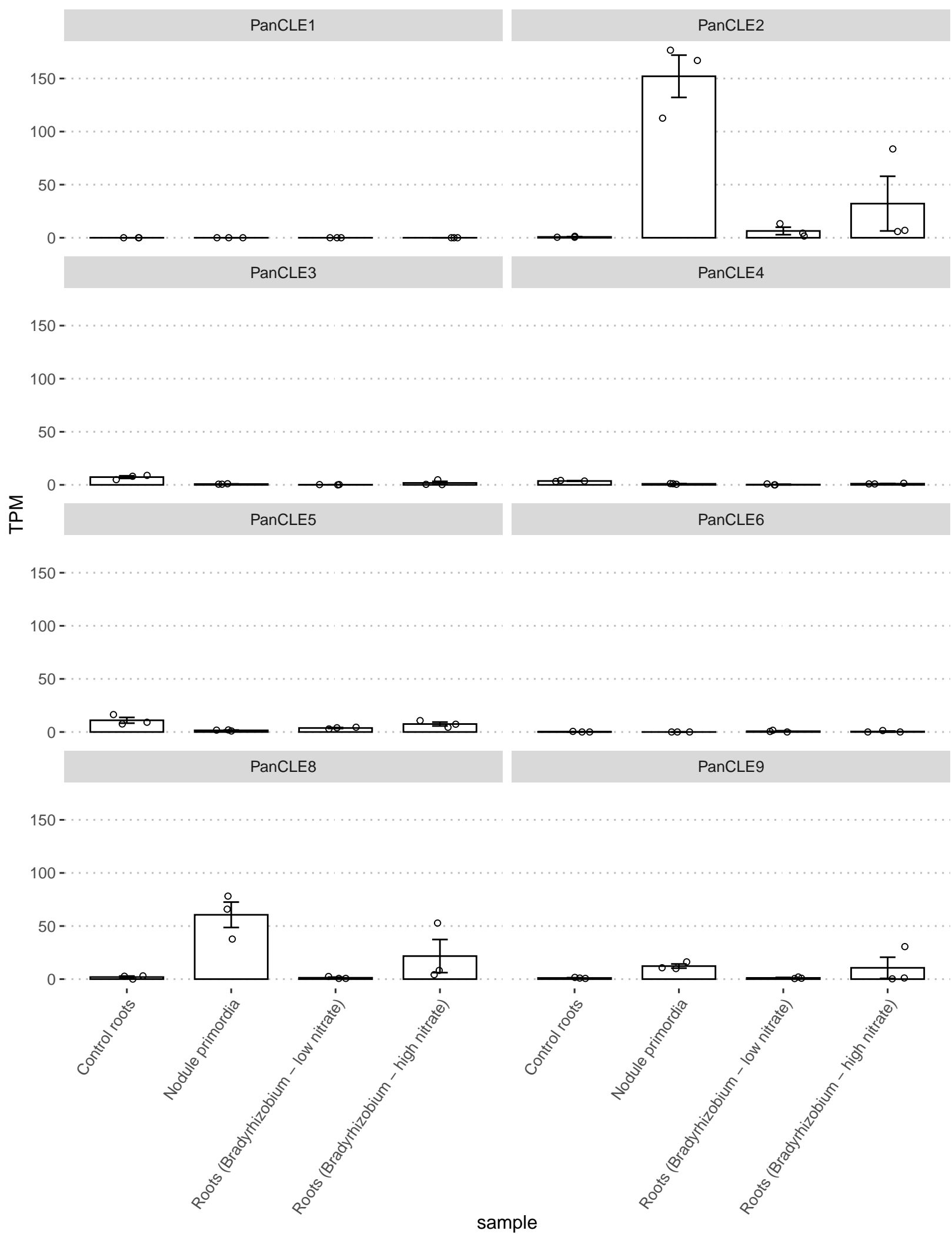

Supplement: Figure S1 — Expression profile of P. andersonii CLE peptide encoding gene. Expression profile of P. andersonii CLE genes in non inoculated roots, nodule promordia and inoculated roots under low (0.5 mM KNO3) and high nitrate (5.0 mM KNO3) conditions. Expression is given in DESeq2-normalized read counts; error bars represent SE of three biological replicates. Dots represent individual expression levels. [file DataSheet_1.pdf]
