# Supplementary material for: The Non-Legume Parasponia andersonii Mediates the Fitness of Nitrogen-Fixing Rhizobial Symbionts Under High Nitrogen Conditions
Source: Front Plant Sci. 2020 Feb 7;10:1779. doi: 10.3389/fpls.2019.01779 (PMC7019102; doi:10.3389/fpls.2019.01779)
Supplement: Supplementary file 2 [file Table_1.docx]

**Table S1**: CLE peptide encoding genes annotated in the *Parasponia andersonii* genome

**Gene name gene ID GenBank notes**

***__________________________________________________________________________________________***

*PanCLE1* PanWU01x14_033260 PON76640 *-*

*PanCLE2* PanWU01x14_022360 PON77882 -

*PanCLE3* PanWU01x14_285420 PON41943 -

*PanCLE4* PanWU01x14_256770 PON45685 -

*PanCLE5* PanWU01x14_285420 PON41943 Homologous to LjCLE-RS1 / LjCLE-RS2

*PanCLE6* PanWU01x14_256780 PON45686 -

*PanCLE8* PanWU01x14_022310 PON77877 -

*PanCLE9* PanWU01x14_285410 PON41942 Homologous to LjCLE-RS1 / LjCLE-RS2

Note: *P. andersonii* doesn’t have a functional *PanCLE7* gene as it has been found in its close relative *Trema orientalis* (Van Velzen et al., 2018).
